# Does the mind map learning strategy facilitate information retrieval and critical thinking in medical students?

**DOI:** 10.1186/1472-6920-10-61

**Published:** 2010-09-16

**Authors:** Anthony V D'Antoni, Genevieve Pinto Zipp, Valerie G Olson, Terrence F Cahill

**Affiliations:** 1 Department of Graduate Programs in Health Sciences, School of Health and Medical Sciences, Seton Hall University, 400 South Orange Avenue, South Orange, NJ 07079, USA; 2 Division of Pre-clinical Sciences, New York College of Podiatric Medicine, 53 East 124th Street, New York, NY 10035, USA

## Abstract

**Background:**

A learning strategy underutilized in medical education is mind mapping. Mind maps are multi-sensory tools that may help medical students organize, integrate, and retain information. Recent work suggests that using mind mapping as a note-taking strategy facilitates critical thinking. The purpose of this study was to investigate whether a relationship existed between mind mapping and critical thinking, as measured by the Health Sciences Reasoning Test (HSRT), and whether a relationship existed between mind mapping and recall of domain-based information.

**Methods:**

In this quasi-experimental study, 131 first-year medical students were randomly assigned to a standard note-taking (SNT) group or mind map (MM) group during orientation. Subjects were given a demographic survey and pre-HSRT. They were then given an unfamiliar text passage, a pre-quiz based upon the passage, and a 30-minute break, during which time subjects in the MM group were given a presentation on mind mapping. After the break, subjects were given the same passage and wrote notes based on their group (SNT or MM) assignment. A post-quiz based upon the passage was administered, followed by a post-HSRT. Differences in mean pre- and post-quiz scores between groups were analyzed using independent samples *t*-tests, whereas differences in mean pre- and post-HSRT total scores and subscores between groups were analyzed using ANOVA. Mind map depth was assessed using the Mind Map Assessment Rubric (MMAR).

**Results:**

There were no significant differences in mean scores on both the pre- and post-quizzes between note-taking groups. And, no significant differences were found between pre- and post-HSRT mean total scores and subscores.

**Conclusions:**

Although mind mapping was not found to increase short-term recall of domain-based information or critical thinking compared to SNT, a brief introduction to mind mapping allowed novice MM subjects to perform similarly to SNT subjects. This demonstrates that medical students using mind maps can successfully retrieve information in the short term, and does not put them at a disadvantage compared to SNT students. Future studies should explore longitudinal effects of mind-map proficiency training on both short- and long-term information retrieval and critical thinking.

## Background

The amount of information that medical students are expected to master is voluminous[1]. Yet, there are limited learning strategies available to these students to master the volume of information required to succeed in medical school[2]. In recent years, the number of publications on learning strategies used in medical education that may help students learn and ultimately integrate information has increased[3-6]. Although these learning strategies may differ in efficacy and applicability, they are all based on a conceptual framework called the constructivist theory of learning, which states that meaningful learning, or learning with understanding, occurs when adult learners assimilate new information within their existing frameworks[7,8].

Constructivist theory is rooted in the subjectivist worldview, which emphasizes the role of the learner within the context of his environment[9]. The interaction between the learner and his environment results in meaning or understanding; therefore, the two are inextricable[9]. Many learning strategies, such as case-based learning and PBL, assume the learner is committed to lifelong learning and will integrate previous knowledge with newly acquired knowledge[10,11].

**Figure 1:**
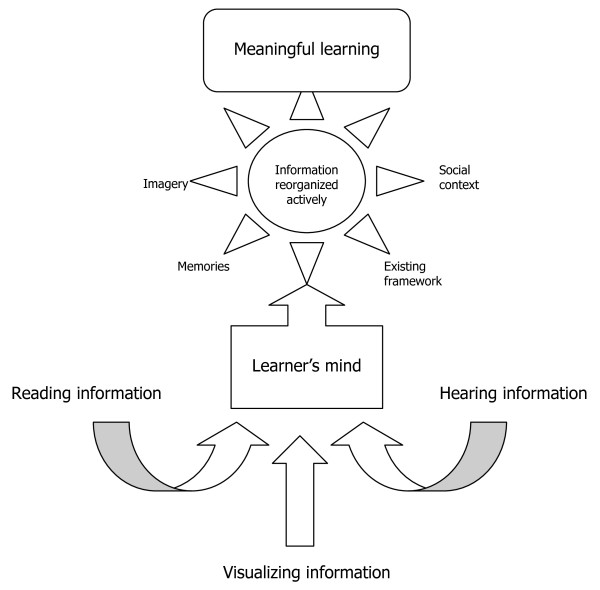
**Constructivist theory of learning**. Theoretical assumptions that underlie constructivist theory using a bottom-up approach. Academic information is commonly available to the learner through reading, visualizing, or listening. Irrespective of the mechanism, information enters the mind of the learner, who is actively trying to make sense of the information. Adapted from Ausubel [7].

The theoretical basis of constructivism is depicted in Figure 1. In medical school, academic information is available to the medical student through reading, visualizing, or listening. Irrespective of the mechanism, information enters the mind of the student, who is actively trying to make sense of the information. Because the sensemaking of the student may be very different from that of the professor presenting the information,[12] one of the assumptions underlying constructivist theory is that the student will integrate the information into a personal framework so that it will be retained,[8] which results in meaningful learning.

### Critical thinking

Meaningful learning is necessary for critical thinking. The operational definition of critical thinking is a metacognitive, nonlinear process of purposeful judgment that includes self-directed learning and self-assessment[13,14]. How critical thinking should be taught and how it is learned are unclear,[15,16] especially at the medical school level. Willingham[15] stated that critical thinking occurs when a student penetrates beyond the surface structure of a problem and recognizes how the problem can be solved, and in addition, possesses the content knowledge integral to solving the problem. Without both components, a student may be able to critically analyze one problem, but will falter when given a similar problem in a different context[15]. Graduating physicians should be able to critically evaluate novel cases that they encounter in the clinic using their previous, albeit limited, clinical experiences[17].

### Concept mapping in medical education

In graduate medical education, West et al[17] used the concept map learning strategy developed by Joseph Novak[18] in resident physicians, and studied the validity and reliability of concept mapping assessment (CMA). They found that concept maps could be scored reliably and CMA could measure changes in the conceptual framework of physicians[17].

### Mind mapping in medical education

Mind mapping was developed by Tony Buzan[19] and the inspiration for this strategy arose from the notebooks of Leonardo da Vinci[20]. Mind maps, like da Vinci's notes, are multi-sensory tools that use visuospatial orientation to integrate information, and consequently, help students organize and retain information[21,22].

Mind maps can be used as a teaching tool to promote critical thinking in medical education by encouraging students (adult learners) to integrate information between disciplines and understand relationships between the basic and clinical sciences[21]. The ability to integrate information by finding valid relationships between concepts allows students who construct either mind maps or concept maps to reach a metacognitive level[15]. However, the added dimensions of pictures and colors that are unique to mind maps have not only been shown to facilitate memory,[23] but may appeal to a wide range of students withvisual- and linear-oriented learning styles. Consequently, the advantage of using mind maps in medical education is that this strategy may benefit more students with diverse learning styles.

Both mind maps and concept maps allow students to recognize the intra- and inter-relationships between concepts, which reflects the kind of real-world thinking predominant in the clinical setting[24].

Farrand et al[25] were the first group to investigate the potential role of mind mapping in medical education. These researchers explored whether the mind map learning technique was superior to traditional note taking in both short- and long-term factual recall of written information in medical students. They found that the mind map technique significantly improved long-term memory of factual information. Additionally, they found significant differences in self-reported motivation with the mind map group having lower levels of motivation than the self-selected study group. Although not supported by other literature, this finding may be explained by the fact that students were not given adequate time to adjust to using the mind map technique, and therefore, may have felt less comfortable using it. Although the results of the study were promising, the authors did not address critical thinking. Consequently, studies exploring the relationship between mind mapping and critical thinking are needed before the usefulness of mind mapping can be fully supported in medical education.

Wickramasinghe et al[26] were the second group to investigatethe effectiveness of mind maps in medical education. Using a similar study design as that used by Farrand et al,[25] these authors assigned new entry medical students into 2 groups: mind map and self-selected study groups. The authors also developed a method to score the mind maps based on structure and content; however, they did not describe the method nor did they provide any data to support it[26]. The authors reported that there was no significant difference in scores between groups[26]. They did, however, report that all of the subjects in the mind map group perceived that mind maps are useful for memorizing information. Based on their findings, the authors concluded that mind mapping may not be effective in improving retention of short-term information[26].

### Mind maps and concept maps

**Figure 2:**
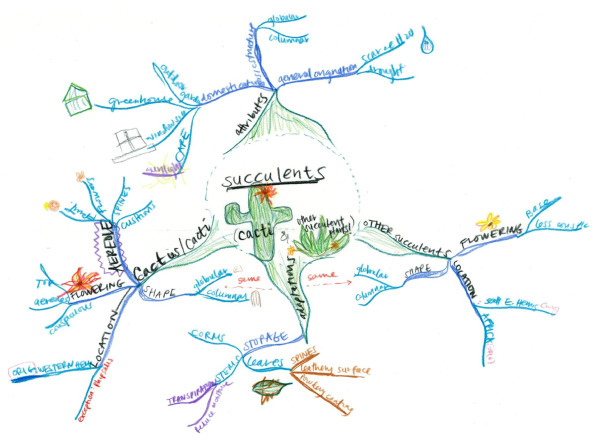
**Student mind map**. An example of a mind map from one of the medical students in this study. Note the judicious use of pictures and colors, along with hierarchical organization positioned radially. Note how different colors were used to indicate different hierarchies (eg, green is primary hierarchy, blue is secondary, aqua is tertiary, etc.). In addition to the above example, other student mind maps have been published elsewhere[22,28].

Although concept maps and mind maps have similar characteristics, they are fundamentally different in design. Concept maps are devoid of color and pictures, and are constructed in a top-to-bottom hierarchy. Mind maps, in contrast, use a central theme in the middle of a page with categories and subcategories that radiate peripherally, thus making them truly non-linear. The cross-links among categories highlight their intrinsic relationships, and allow the student to compare and contrast information. Unlike concept maps, mind maps are multisensory--they include color and pictures, which facilitate the conversion of information from short- to long-term memory[23,27]. An example of a mind map created by a medical student in this study can be found in Figure 2.

Since critical thinking is dependent upon both content (domain) knowledge and problem familiarity,[15] mind mapping may facilitate critical thinking because it fosters student retention of factual information, as well as relationships between concepts[25]. Currently, however, there are no data to support the hypothesis that mind maps facilitate critical thinking in medical students.

### Purpose of the study

The primary purpose of this study was to investigate whether a relationship existed between the mind map learning strategy and critical thinking, as measured with the Health Sciences Reasoning Test (HSRT), and whether this relationship was stronger than one between the preferred learning strategy of standard note-taking (SNT) and critical thinking.

The secondary purpose of this study was to determine whether mind maps were superior to SNT in the short-term recall of factual information. Mind map depth was assessed using the previously published Mind Map Assessment Rubric[28].

## Methods

### Study setting and sample

After full approval by an Institutional Review Board, this study was conducted during the 2008-2009 academic year at a US medical school located in a large metropolitan area.

An *a priori *power analysis[29] using a one-tailed *t*-test revealed a minimum sample size of 70 subjects. This calculation was based on the following: effect size d = 0.8, alpha = 0.05, and power = 0.95. The large sample size (*N *= 131) assumes a normal distribution of the population, and therefore, parametric statistics were used to analyze the data. The sample of convenience consisted of first-year medical students who voluntarily participated in this study.

### Procedures

**Figure 3:**
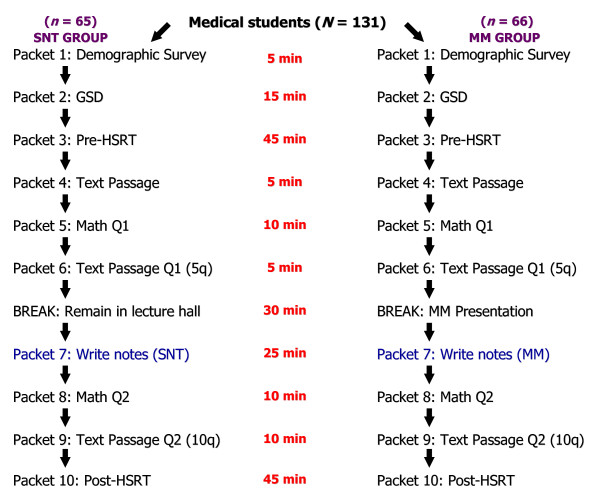
**Study design**. Research procedure.

The independent variable in this study was the note-taking strategy used by the medical students. Subjects were randomly assigned to 2 note-taking groups: a standard note-taking (control) group and mind map (experimental) group. The design of the study is outlined in Figure 3.

Subjects in both note-taking groups were asked to learn information contained in a 394-word text passage—on the topic of cacti and other succulent plants—from the verbal ability section of a previously published Graduate Record Examination (GRE). This topic was chosen to reduce the chance that the medical students would have previous advanced knowledge of this field. The GRE is a standardized entrance examination used as part of the US graduate-school admissions process. The exam is used by faculty to decide which students will be admitted to graduate school and who will be awarded academic fellowships. A GRE text passage was used in this study because the GRE is taken by students who are, in general, of a similar age to those entering US medical schools. Consequently, the text passage was at an appropriate cognitive level for medical students. A *post hoc *analysis of the medical students in the study revealed that none of them majored in botany at the undergraduate level.

Subjects in the control group used standard note-taking (SNT) strategies that they used throughout their academic careers to learn the text passage. SNT is defined as any study strategy that does not rely on reorganizing information using architecture commonly seen in a concept map or mind map[25]. SNT is a process whereby notes are arranged in a hierarchy from the top of a page to the bottom, or from left to right, without any hierarchy[30]. Subjects in the experimental (mind map) group were given a 30-minute presentation on mind maps and then instructed to create mind maps in order to take notes on the material in the text passage.

There were two dependent variables in this study. The first one was the score on the text passage quiz, of which there were two. These two quizzes, which were based on the content of the GRE text passage, were administered to all subjects after assignment to the groups. All subjects were simultaneously (but in different rooms) exposed to the passage for 5 minutes and were not permitted to write any notes. The passage was collected and followed by the administration of math quiz 1. This quiz was used to "blank" the minds of the subjects by preventing the simple recall of information that could result in a higher quiz score and confound the results[25].

After math quiz 1, all subjects were administered text passage quiz 1. The purpose of this 5 multiple-choice question quiz was to test the students' factual understanding of the passage without any note-taking strategy. This baseline quiz was used as a covariate to account for potential differences between the groups prior to initiating any note-taking strategy.

After taking text passage quiz 1, subjects in the mind map group were given a presentation on mind maps and how to construct them, while at the same time, subjects in the control group were sequestered for a break and could not leave the lecture hall. After 30 minutes, all subjects were then re-exposed to the text passage and instructed to take notes using either standard note-taking (SNT) or mind maps (MMs), depending on their group assignment. All subjects were given 25 minutes for note-taking and at the end of this time period, all passages and notes were collected. This was followed by the administration of math quiz 2 in order to again discourage the simple recall of information by the subjects. After math quiz 2, all subjects were simultaneously administered text passage quiz 2 based upon the passage. This quiz consisted of 10 multiple-choice questions: the same 5 questions from quiz 1 plus an additional 5 questions. This was done to see if the students retained the factual information and to address potential testing effects (ie, higher scores due to repeated testing exposure).

The second dependent variable of this study was the HSRT score. The HSRT consists of 33 multiple-choice questions that measure critical thinking by challenging students to form reasoned judgments based on textually presented information consisting of a number of vignettes[31]. The information presented in the vignettes includes diagrams, charts, and other data related to health care scenarios. The HSRT does not test domain knowledge (ie, subject-specific knowledge such as that found in anatomy and biochemistry); therefore, subject-specific knowledge is not needed by the students taking the exam. The HSRT has been extensively studied in health professional students and working professionals[14,31].

The HSRT reports an overall numerical score and 5 subscales: analysis, inference, evaluation, deductive reasoning, and inductive reasoning. The operational definitions of these subscales, adapted from a previous Delphi study, [14] follow: analysis (ability to identify the intended and actual inferential relationships among statements, questions, concepts, descriptions or other forms of representation intended to express beliefs, judgments, experiences, reasons, information or opinions); inference (ability to identify and secure elements needed to draw reasonable conclusions; to form conjectures and hypotheses, to consider relevant information and to educe the consequences flowing from data, statements, principles, evidence, judgments, beliefs, opinions, concepts, descriptions, questions, or other forms of representation); evaluation (ability to state the results of one's reasoning; to justify that reasoning in terms of the evidential, conceptual, methodological, criteriological and contextual considerations upon which one's results were based; and to present one's reasoning in the form of cogent arguments); deductive reasoning (assumed truth of the premises purportedly necessitates the truth of conclusion and this includes traditional syllogisms, as well as, algebraic, geometric, and set-theoretical proofs in mathematics); and inductive reasoning (an argument's conclusion is purportedly warranted, but not necessitated, by the assumed truth of its premises and this includes scientific confirmation and experimental disconfirmation)[31].

Mind maps were scored using the Mind Map Assessment Rubric (MMAR). The interrater reliability of the MMAR is strong and has been reported to be 0.86[28]. Face validity of the MMAR has been investigated, and the entire rubric is available online (see reference [28]).

## Results

### Sample characteristics

A total of 131 subjects (*N *= 131) participated in the study (Table 1). All subjects were matriculated, first-year medical students and the study was conducted on a half-day during their orientation. Prior to the study, subjects were queried and it was found that none of them used mind maps as their preferred learning strategy. The SNT group consisted of 65 subjects (*n *= 65) and the MM group consisted of 66 subjects (*n *= 66).

**Table 1:** Demographic comparison between subjects in both groups (*N *= 131)

		SNT Group (*n *= 65)	MM Group (*n *= 66)
Gender	Male	32 (49.2%)^a^	31 (47.0%)
	Female	33 (50.8%)	35 (53.0%)
			
		**SNT Group (*n *= 64)**^**b**^	**MM Group (*n *= 64)**^**c**^
Ethnicity	African American	1 (1.6%)	3 (4.7%)
	Anglo American, Caucasian	29 (45.3%)	35 (54.7%)
	Asian American/Pacific Islander	23 (35.9%)	18 (28.1%)
	Hispanic, Latino, Mexican American	1 (1.6%)	3 (4.7%)
	Mixed/Other	10 (15.6%)	5 (7.8%)

^a^Data are presented as number of subjects (percentage) within the group. ^b^One subject in the control group did not disclose ethnicity. ^c^Two subjects in the study group did not disclose ethnicity.

Sex and ethnicity distributions were similar in both groups as demonstrated in Table 1. The mean age of subjects in both groups was also similar. In the SNT group, the mean age of subjects was 24.45 years (*SD *= 3.26) and in the MM group, the mean age of subjects was 24.74 years (*SD *= 3.91). Using one-way analysis of variance (ANOVA), no significant difference in mean age between groups was found. Subjects in the SNT group had a mean total SAT score of 1285.71 (*SD *= 112.06) and those in the MM group had a mean total SAT score of 1254.46 (*SD *= 110.20). No significant difference in total SAT score between groups was found. In addition, no significant differences in SAT verbal and math subscores between groups were found. The mean total MCAT score of subjects in the SNT group was 27.26 (*SD *= 3.04) and the mean total MCAT score of subjects in the MM group was 27.05 (*SD *= 3.17). No significant difference in total MCAT score between groups was found. In addition, no significant differences in MCAT biology, physics, and verbal subscores between groups were found.

### Quiz assessment of domain knowledge

The mean score of the pre-quiz (quiz 1) among subjects in the SNT group was 3.15 (*SD *= 1.22) and the mean score of the pre-quiz (quiz 1) among subjects in the MM group was 3.42 (*SD *= .84). A two-tailed independent samples *t *test revealed no significant difference between the means: *t *(129 df) = -1.47, *p *= .14.

**Figure 4:**
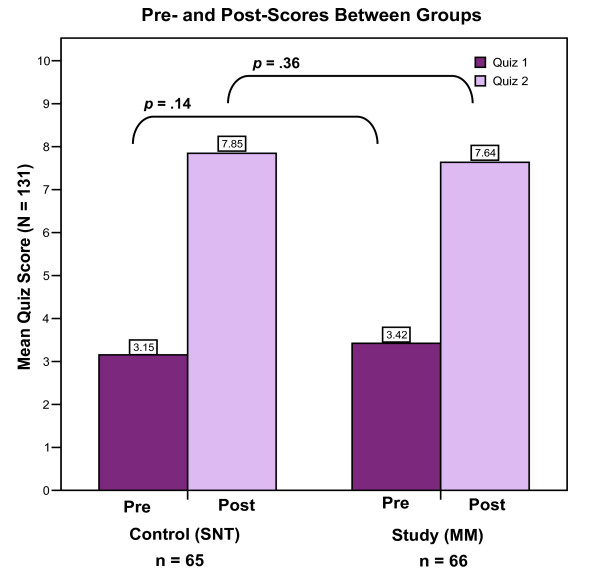
**Quiz scores between groups**. Both quizzes were based on a 394-word text passage. There are no significant differences in mean scores between groups on both the pre-quiz (quiz 1) and post-quiz (quiz 2).

The mean score of the post-quiz (quiz 2) among subjects in the SNT group was 7.85 (*SD *= 1.40) and the mean score of the post-quiz (quiz 2) among subjects in the MM group was 7.64 (*SD *= 1.22). A two-tailed independent samples *t *test revealed no significant difference in means between the groups: *t *(129 df) = .912, *p *= .36. Figure 4 is a bar chart depicting these data.

A comparison of the means of the pre-quiz (quiz 1) scores and post-quiz (quiz 2) scores between groups revealed no significant differences (SNT pre-quiz mean = 3.15, MM pre-quiz mean = 3.42, SNT post-quiz mean = 7.85, and MM post-quiz mean = 7.64). However, the difference between means of the pre-quiz (quiz 1) and post-quiz (quiz 2) scores in each group differed. In the SNT group, this difference was 4.70 (7.85 - 3.15 = 4.70) and in the MM group, this difference was 4.22 (7.64 - 3.42 = 4.22).

**Figure 5:**
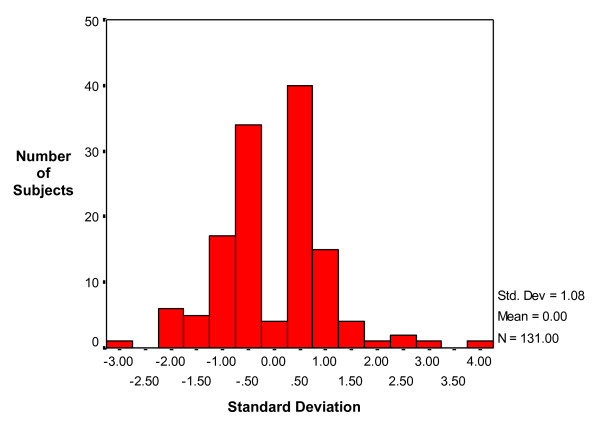
**Differences between quiz scores using a standardized *z *score**. A difference score was created between the standardized quiz scores so that the degree to which the variability in each quiz affected the outcome would be the same. The difference score is standardized with a mean of 0 and a *SD *of 1.08. On the average, subjects in the MM group had lower scores on the second quiz (-.2061 *SD*), while those in the SNT group increased by about the same amount (.2093 *SD*). This represents about two-tenths of a *SD *and the difference was found to be significant (*p *= .027).

In order to further analyze these results and control for the fact that the quiz scores themselves were slightly skewed (ie, a long tail created by a few students who did very poorly), a standardized *z *score was used. A difference *z *score was created between the standardized quiz scores so that the degree to which the variability in each quiz affected the outcome would be the same. Unlike the quiz scores, the difference *z *score conforms to a Gaussian distribution as demonstrated in Figure 5. The difference *z *score is standardized with a mean of 0 and a *SD *of 1.08. On the average, subjects in the MM group had lower scores on the second quiz (-.2061 *SD*), while those in the SNT group increased by about the same amount (.2093 *SD*). This represents about two-tenths of a *SD*. The fact that the scores of the groups vacillated by almost the same amount is not by chance. A two-tailed independent samples *t *test revealed a significant difference between the means of the *z *score difference: *t *(129 df) = 2.241, *p *= .027.

### HSRT assessment of critical thinking

Descriptive statistics of pre-HSRT scores for all subjects (*N *= 131) were as follows: total (*M *= 23.75, *SD *= 3.38), analysis (*M *= 4.85, *SD *= 1.06), inference (*M *= 3.82, *SD *= 1.25), evaluation (*M *= 5.30, *SD *= .84), induction (*M *= 7.97, *SD *= 1.20), and deduction (*M *= 7.59, *SD *= 1.76). Descriptive statistics of post-HSRT scores for all subjects (*N *= 131) were as follows: total (*M *= 23.73, *SD *= 3.78), analysis (*M *= 4.84, *SD *= 1.05), inference (*M *= 3.74, *SD *= 1.24), evaluation (*M *= 5.28, *SD *= .88), induction (*M *= 7.96, *SD *= 1.24), and deduction (*M *= 7.69, *SD *= 1.91). Descriptive statistics comparing pre-HSRT scores between subjects in the SNT group and MM group are found in Table 2. Similarly, descriptive statistics comparing post-HSRT scores between subjects in the SNT group and MM group are found in Table 3.

**Table 2:** Descriptive statistics of pre-Health Sciences Reasoning Test (pre-HSRT) scores in SNT and MM groups (*N *= 131)

Variable	*M*	*Mdn*	*Trimmed* *M*	*SD*	*SEM*	*Min*^a^	*Max*^b^
SNT Group (*n *= 65)							
Total Score	23.41	24	23.54	3.69	.45	11	31
							
Subscale Scores^c^							
Analysis	4.72	5	4.81	1.21	.15	1	6
Inference	3.78	4	3.81	1.30	.16	1	6
Evaluation	5.27	5	5.37	.89	.11	2	6
Inductive Reasoning	7.98	8	8.10	1.26	.15	3	10
Deductive Reasoning	7.43	8	7.57	1.97	.24	2	10

MM Group (*n *= 66)							
Total Score	24.07	24	24.05	3.04	.37	16	33
							
Subscale Scores^c^							
Analysis	4.98	5	5.03	.88	.10	3	6
Inference	3.86	4	3.88	1.21	.14	1	6
Evaluation	5.31	5	5.38	.80	.09	2	6
Inductive Reasoning	7.95	8	7.98	1.14	.14	5	10
Deductive Reasoning	7.74	8	7.76	1.52	.18	5	10

^a^Minimum. ^b^Maximum. ^c^There are five HSRT subscales: analysis, inference, evaluation, inductive reasoning, and deductive reasoning.

**Table 3:** Descriptive statistics of post-Health Sciences Reasoning Test (post-HSRT) scores in SNT and MM groups (*N *= 131)

Variable	*M*	*Mdn*	*Trimmed* *M*	*SD*	*SEM*	***Min***^a^	***Max***^b^
SNT Group (*n *= 65)							
Total Score	23.47	24	23.66	3.82	.47	9	30
							
Subscale Scores^c^							
Analysis	4.87	5	4.94	1.05	.13	1	6
Inference	3.72	4	3.74	1.26	.15	1	6
Evaluation	5.24	6	5.35	1.03	.12	2	6
Inductive Reasoning	7.96	8	8.05	1.26	.15	4	10
Deductive Reasoning	7.58	8	7.74	2.06	.25	1	10

MM Group (*n *= 66)							
Total Score	23.97	24	24.20	3.75	.46	12	30
							
Subscale Scores^c^							
Analysis	4.80	5	4.88	1.05	.13	1	6
Inference	3.75	4	3.76	1.22	.15	1	6
Evaluation	5.31	5	5.36	.72	.08	3	6
Inductive Reasoning	7.95	8	8.01	1.24	.15	4	10
Deductive Reasoning	7.78	8	7.90	1.75	.21	2	10

^a^Minimum. ^b^Maximum. ^c^There are five HSRT subscales: analysis, inference, evaluation, inductive reasoning, and deductive reasoning.

**Figure 6:**
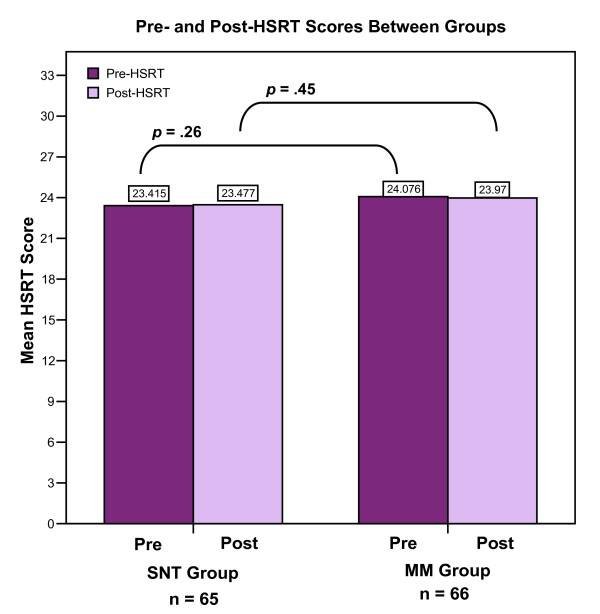
**HSRT total scores between groups**. There are no significant differences in mean total scores between groups on both the pre-HSRT and post-HSRT.

ANOVA was used to compare the means of pre- and post-HSRT total scores and subscores between the SNT group and MM group. No significant differences were found among any of the pre- and post-HSRT total scores and subscores. The bar chart in Figure 6, which displays pre- and post-HSRT total scores, demonstrates no significant differences between pre- and post-HSRT total scores between groups.

## Discussion

The difference in mean score of the pre-quiz (quiz 1) between subjects in the SNT group and MM group was not significant. This baseline finding suggests that both groups retained the same amount of information equally based upon a single, 5-minute exposure to the text passage.

The post-quiz (quiz 2) was administered to subjects after they were re-exposed to the text passage and instructed to write notes using either their preferred note-taking strategy (SNT) or newly acquired mind mapping (MM) strategy. Although the mean score of the post-quiz (quiz 2) was slightly higher among subjects in the SNT group (7.85, *SD *= 1.40) compared to those in the MM group (7.64, *SD *= 1.22), the difference was not significant. This result suggests that mind mapping is not superior to standard note-taking for the short-term recall of domain-based information, an outcome that concurs with the results of Wickramasinghe et al.[26]. However, it should be emphasized that subjects in the MM group did not score significantly less than those in the SNT group even though they were only given a single, brief overview of the mind map learning strategy without a practice period to increase proficiency in creating mind maps. The fact that no significant difference was found between groups may lend support to the utility of mind mapping in medical education. Subjects in the SNT group had the benefit of using their preferred note-taking strategy and by allowing them to do so, these subjects were able to cognitively organize, integrate, and learn the information based on a system that has been firmly reinforced throughout their academic careers. A *post hoc *analysis of the notes written by SNT subjects revealed that none of them wrote notes remotely similar to mind maps or concept maps. In fact, most of their notes were written in a traditional categorical way with information starting at the top of the page and ending at the bottom. Consequently, subjects in the SNT group focused on learning the material in a short period of time without being distracted to write notes in a new way. In contrast, subjects in the MM group were forced to use the unfamiliar mind map learning strategy (based on a brief introductory learning session) that may have distracted them from optimally learning the material. Yet, despite the lack of exposure to mind maps and their novice status, subjects in the MM group were able to integrate, and ultimately, retain enough information so that they did not score significantly less than subjects in the SNT group. This important finding suggests the strength of mind mapping even after a single, 30-minute introductory session in promoting critical thinking in the novice learner, and supports the notion of adult learner capability[7].

As mentioned previously, there were 10 questions on quiz 2: the first 5 were the same questions found on quiz 1 and questions 6 through 10 were new. When looking at questions 6 through 10 on quiz 2, the mean score among subjects in the SNT group was 3.95 (*SD *= .87) and the mean score among subjects in the MM group was 3.79 (*SD *= .86). This difference was not found to be significant. Similar to responses for questions 1 through 5 on quiz 2, the mean score in the SNT group was slightly higher on quiz 2 (questions 6 through 10) than the MM group, but not significant. Again, this finding may have been due to the fact that subjects in the SNT group were using a familiar note-taking strategy, whereas those in the MM were using an unfamiliar strategy.

Further analysis of the difference between mean total scores of the pre-quiz (quiz 1) and post-quiz (quiz 2) in each group was calculated using a standardized *z *score (Figure 6). The SNT group revealed an increase of about two-tenths of a *SD *(.2093 *SD*), while the MM group decreased by about two-tenths of a *SD *(-.2061 *SD*). Using a two-tailed independent samples *t *test, this difference was found to be significant . This result suggests that mind mapping did not enhance short-term memory in this novice group of subjects who were only exposed to a brief overview of how to construct mind maps.

The results of the present study support those of Wickramasinghe et al,[26] who found that the mean quiz score of subjects in their mind map group was 31.3% and the mean quiz score of subjects in their self-selected study group was 37.6%. These authors reported that there was no significant difference in scores between groups[26]. However, the results of the present study are in contrast to those of Farrand et al,[25] who reported that recall was only slightly higher in the mind map group after the second quiz. After adjusting for baseline performance and motivation, this difference was significant. Without the adjustment, the difference was not significant, which is consistent with the findings of the present study. Farrand et al[25] reported a robust difference in recall in favor of subjects in the mind map group after one week.

### HSRT assessment of critical thinking

The mean total score on the pre-HSRT for subjects in the SNT group was 23.41 (*SD *= 3.69) and the mean total score on the pre-HSRT for subjects in the MM group was 24.07 (*SD *= 3.04). This difference was not significant and this finding demonstrates that both groups had similar baseline critical thinking abilities as measured by the HSRT.

The mean total score on the post-HSRT for subjects in the SNT group was 23.47 (*SD *= 3.82) and the mean total score on the post-HSRT for subjects in the MM group was 23.97 (*SD *= 3.75). Subjects in the MM group did not score significantly different than those in the SNT group on the post-HSRT, a finding that suggests the power of mind mapping even when it was introduced to a novice group of subjects during a brief introductory session. The fact that subjects in the MM group scored worse on the post-HSRT compared to their pre-HSRT total scores could be explained by their unfamiliarity in creating mind maps or fatigue from the testing process. Additionally, requiring MM subjects to learn mind mapping may have created contextual interference that hampered short-term retention as demonstrated by the results of the post-HSRT; however, this may actually promote long-term retention as noted in the contextual interference literature[32]. Subjects in the MM group may have been so preoccupied with creating mind maps that they failed to think critically about the information. Therefore, repeated exposure to mind mapping over time may be a necessary requisite in order to better test whether the use of mind mapping increases critical thinking as measured by the HSRT.

### Limitations and future research

The SNT group remained in the lecture hall during the break while the MM group was concomitantly exposed to a 30-minute mind map presentation. A potential limitation, therefore, is that during the break subjects in the SNT group could have mentally reviewed the text passage. These subjects were observed during this time and were not permitted to view the text passage. The possibility that they were able to accurately recall the text passage during the break (while the MM group listened to the presentation) is unlikely because they were exposed to the text passage 20 minutes before the break and had also taken an intervening math quiz (see Figure 3).

Because critical thinking takes time to develop, short-term changes in critical thinking was another limitation of the current study. Multiple mind-map sessions may be necessary for students to gain proficiency in the strategy before significant changes in the acquisition of domain-based knowledge and critical thinking emerge. Recently, Srinivasan et al[24] reported that concept map scores significantly increased in physicians who created concept maps on two separate occasions. They recommended that future concept map studies should allow subjects to create concept maps on multiple occasions. This may also be true of mind maps because, although not investigated in medical students, researchers have demonstrated that mind map depth increases as students gain proficiency in their construction over time[13,30].

Future studies should be designed to allow subjects to create multiple mind maps so that they can gain proficiency in the technique. This would enable them to move from novice to expert regarding the creation of mind maps, and therefore, could ultimately allow them to emphasize critical thinking. Additionally, these studies could also measure longitudinal changes in HSRT scores as students become more proficient at mind mapping.

## Conclusions

The results of this study demonstrate that the mind map learning strategy does not result in a significant gain in short-term, domain-based knowledge (assessed using multiple-choice quizzes) compared to standard note-taking in medical students. However, in subjects who were unfamiliar with mind mapping, a short 30-minute presentation on the strategy allowed them to score similarly to subjects in the SNT group who used strategies that have been firmly established. By using preferred note-taking strategies, subjects in the SNT group were able to rely on previous note-taking experiences that helped shaped their current understanding and learning of the material in the text passage,[10] while those in the MM group could not rely on prior mind map note-taking experiences as they were novices. Subjects in the MM group may have relied on previous knowledge of other non-mind map note-taking strategies, which could explain why they were able to score similarly. The similarity in mean scores between groups lends support to adult learning theory[7,8,11].

This study demonstrates that mind mapping can be easily taught to medical students who have no previous background in mind mapping and doing so requires no cost or expensive equipment [22,33]. Thus, mind mapping may be an attractive resource to add to the study-strategy repertoire of entering medical students to help them learn and organize information. As discussed by Daley and Torre [34] in a recent analytical review, the effects of mapping need to be investigated longitudinally. The data of the present study build upon those of previous studies [25,26] and should provide a springboard for those interested in investigating the effect of mind mapping on critical thinking and clinical reasoning during medical school and beyond.

## Abbreviations

ANOVA: analysis of variance; CM: concept map; CMA: concept map assessment; GRE: Graduate Record Examination; HSRT: Health Sciences Reasoning Test; *M*: mean; *Max*: maximum; MCAT: Medical College Admissions Test; *Mdn*: median; *Min*: minimum; MM: mind map; MMAR: mind map assessment rubric; PBL: problem-based learning; *SD*: standard deviation; *SEM*: standard error of the mean; SNT: standard note-taking

## Competing interests

The authors declare that they have no competing interests.

## Authors' contributions

AVD conceived the design of the study, performed the statistical analyses, scored the mind maps, and drafted the manuscript. GPV participated in the design of the study, scored the mind maps, and drafted the manuscript. VGO participated in the design of the study, scored the mind maps, and helped draft the manuscript. TFC participated in the design of the study and helped draft the manuscript. All authors read and approved the final manuscript.

## Pre-publication history

The pre-publication history for this paper can be accessed here:

http://www.biomedcentral.com/1472-6920/10/61/prepub

## References

[B1] Anderson J Graham A A problem in medical education: Is there an information overload? Med Educ 1980 14 4 7 10.1111/j.1365-2923.1980.tb02604.x 7366494

[B2] Rye PD Wallace J Bidgood P Instructions in learning skills: An integrated approach Med Educ 1993 27 470 473 10.1111/j.1365-2923.1993.tb00305.x 8208152

[B3] Barrows HS Practice-based Learning: Problem-based Learning Applied to Medical Education 1994 Springfield: Southern Illinois University School of Medicine

[B4] Dolmans DH De Grave W Wolfhagen IH van der Vleuten CP Problem-based learning: Future challenges for educational practice and research Med Educ 2005 39 732 741 10.1111/j.1365-2929.2005.02205.x 15960794

[B5] Kim S Phillips WR Pinsky L Brock D Phillips K Keary J A conceptual framework for developing teaching cases: A review and synthesis of the literature across disciplines Med Educ 2006 40 867 876 10.1111/j.1365-2929.2006.02544.x 16925637

[B6] Zajaczek JE Gotz F Kupka T Behrends M Haubitz B Donnerstag F Rodt T Walter GF Matthies HK Becker H eLearning in education and advanced training in neuroradiology: Introduction of a web-based teaching and learning application Neuroradiology 2006 48 640 646 10.1007/s00234-006-0108-x 16819653

[B7] Ausubel DP Educational Psychology: A Cognitive View 1978 New York, NY: Holt Rinehart and Winston

[B8] Bodner GM Constructivism: A theory of knowledge J Chem Educ 1986 63 873 878 10.1021/ed063p873

[B9] Burrell G Morgan G Sociological Paradigms and Organisational Analysis 1979 London, England: Heinemann

[B10] Forrest SP III Peterson TO It's called andragogy Acad Manag Learn Educ 2006 5 113 122

[B11] Knowles M The Modern Practice of Adult Education: Andragogy Versus Pedagogy 1977 New York, NY: Association Press

[B12] Mezirow J A critical theory of adult learning and education Adult Educ Q 1981 32 3 24 10.1177/074171368103200101

[B13] Daley BJ Shaw CR Balistrieri T Glasenapp K Piacentine L Concept maps: A strategy to teach and evaluate critical thinking J Nurs Educ 1999 38 42 47 9921788 10.3928/0148-4834-19990101-12

[B14] APA Critical Thinking: A Statement of Expert Consensus for Purposes of Educational Assessment and Instruction ERIC document: ED 1990 315 423

[B15] Willingham DT Critical thinking: Why is it so hard to teach? Am Educator 2007 31 8 19

[B16] Taconis R Ferguson-Hessler MGM Broekkamp H Teaching science problem solving: An overview of experimental work J Res Sci Teach 2001 38 442 468 10.1002/tea.1013

[B17] West DC Pomeroy JR Park JK Gerstenberger EA Sandoval J Critical thinking in graduate medical education: A role for concept mapping assessment? JAMA 2000 284 1105 1110 10.1001/jama.284.9.1105 10974689

[B18] Novak JD Gowin DB Learning How to Learn 1984 Cambridge, England: Cambridge University Press

[B19] Buzan T Buzan B The Mind Map Book 1993 London, England: BBC Books

[B20] Gelb MJ How to Think Like Leonardo da Vinci: Seven Steps to Genius Every Day 1998 New York, NY: Dell

[B21] McDermott P Clarke DN Mind Maps in Medicine 1998 Edinburgh, UK: Churchill Livingstone

[B22] D'Antoni AV Pinto Zipp G Applications of the mind map learning technique in chiropractic education: A pilot study and literature review Journal of Chiropractic Humanities 2006 13 2 11

[B23] Day JC Bellezza FS The relation between visual imagery mediators and recall Mem Cogn 1983 11 251 257 10.3758/bf03196971 6621340

[B24] Srinivasan M McElvany M Shay JM Shavelson RJ West DC Measuring knowledge structure: Reliability of concept mapping assessment in medical education Acad Med 2008 83 1196 1203 10.1097/ACM.0b013e31818c6e84 19202500

[B25] Farrand P Hussain F Hennessy E The efficacy of the 'mind map' study technique Med Educ 2002 36 426 431 10.1046/j.1365-2923.2002.01205.x 12028392

[B26] Wickramasinghe A Widanapathirana N Kuruppu O Liyanage I Karunathilake I Effectiveness of mind maps as a learning tool for medical students South East Asian J Med Educ 2007 1 30 32

[B27] Bellezza FS The spatial arrangement mnemonic J Educ Psychol 1983 75 830 837 10.1037/0022-0663.75.6.830

[B28] D'Antoni A Pinto Zipp G Olson V Interrater reliability of the mind map assessment rubric in a cohort of medical students BMC Med Educ 2009 9 19 10.1186/1472-6920-9-19 19400964 PMC2683832

[B29] Faul F Erdfelder E GPOWER: A priori, post-hoc, and compromise power analysis for MS-DOS computer program 1992 3 Bonn, Germany: Bonn University Department of Psychology

[B30] Pinto Zipp G Maher C D'Antoni AV Mind Maps: Useful schematic tool for organizing and integrating concepts of complex patient care in the clinic and classroom J Coll Teaching Learning 2009 6 59 68

[B31] Facione NC Facione PA The Health Sciences Reasoning Test: Test Manual 2006 Millbrae: The California Academic Press

[B32] Lee TD Magill RA Locus of contextual interference J Exp Psychol Learn 1983 9 730 746 10.1037/0278-7393.9.4.730

[B33] Degirmenci Ü Kreil S Burk S Breuer G Kornhuber J Weih M Anteil stigma-assoziierter themen im psychiatrie-konzept von medizinstudierenden in der einführung in die klinische medizin: Eine mind-map studie GMS Zeitschrift für Medizinische Ausbildung 2010 27 1 5

[B34] Daley BJ Torre DM Concept maps in medical education: An analytical literature review Med Educ 2010 44 440 448 10.1111/j.1365-2923.2010.03628.x 20374475

